# Diseased Children

**Published:** 1911-11

**Authors:** J. N. Hurty

**Affiliations:** Secretary Indiana State Board of Health


For Texas Medical Journal.

### Diseased Children.

DR. J. N. HURTY, SECRETARY INDIANA STATE BOARD OF HEALTH.

A weak, sickly child is indeed a sad sight. The putty complexion, the lackluster eyes, the thin hands, arms and legs, and the weary look makes the heart bleed. But why is the diseased child? How came it to be diseased? Have the sins of the father descended? If they have, why is not he arrested and punished? If he were slowly to poison the child with poison bought at the drug store he would be arrested and punished promptly. What is the difference? Ask the child which poisoning he prefers. He will certainly tell you, after he has suffered and salved his sores for a few years, that arsenic poison is preferable to blood poisoning.

Why does not society class as disgraced him who bears hereditary poison in his blood, having wickedly put it there? And what a strange inconsistent thing is society anyhow. It has one standard of morals for women, and another for men. And so long as this condition prevails, so long will the blood sins of husbands descend upon their wives and children. In the orphans' home at Indianapolis are seventeen innocent children all suffering from the hereditary malady which is worse than leprosy. They can not develop into strong, healthy, useful members of society; the disease prevents. They will be a burden to themselves and to the State all their lives, and possibly they will produce more like themselves. Why does society permit such conditions? We strive to prevent fire, for it destroys property. Why not strive to prevent fire (disease) that burns up human beings? Is it our high intelligence which keeps us silent and inactive in this matter?

The law should require the prompt reporting of the cases of the hereditary plagues. They are, excepting certain instances, acquired in sin and self-disgrace. Why should we speak of the matter in a whisper?

Is our silence strength, or is it weakness?

